# The *Lck* Paradox: Because of Inconsistent Experimental Evidence, the Role of the Protein Tyrosine Kinase p^56^^lck^ in Early Thymic Development Remains Poorly Defined

**DOI:** 10.1155/1998/25758

**Published:** 1998

**Authors:** Klaus Eichmann

**Affiliations:** Max-Planck-Institut für Immunbiologie Stübeweg 51 Freiburg D-79108 Germany


*Developmental Immunology*, 1998, Vol. 6, pp. 19–24
Reprints available directly from the publisher
Photocopying permitted by license only

© 1998 OPA (Overseas Publishers Association)
N.V. Published by license under the
Harwood Academic Publishers imprint,
part of the Gordon and Breach Publishing Group
Printed in Malaysia

### *Minireview*

### The *Lck* Paradox: Because of Inconsistent Experimental Evidence, the Role of the Protein Tyrosine Kinase p56$^{lck}$ in Early Thymic Development Remains Poorly Defined

KLAUS EICHMANN

*Max-Planck-Institut für Immunbiologie, Stübeweg 51, D-79108 Freiburg, Germany*

*(Received 15 September 1996; In final form 14 April 1997)*

Since this article was written, publications by several groups of investigators shed additional light on the *lck* paradox: Firstly, as considered in this mini-review, two papers now show that *fyn* can partially substitute for *lck* in pre-T cell receptor dependent proliferation and differentiation. (T. Groves, P. Smiley, M.P. Cooke K. Forbush, R.M. Perlmutter, and C.J. Guidos. (1996). *fyn* can partially substitute for *lck* in T lymphocytes development. Immunity 5: 417; N.S.C. van Oers, B. Lowin-Kropf, D. Finlay, K. Connolly, and A. Weiss. (1996). $\alpha\beta$ T cell development is abolished in mice lacking both *lck* and *fyn* protein tyrosine kinases. Immunity 5: 429). This work implies that the dominant negative mutant of *lck* competes with both *lck* and *fyn* and thus partially resolves the lck paradox. Secondly, concerning TCR$\beta$ locus allelic exclusion, it was shown that components of the CD3 complex are necessary for the exclusion of endogenous TCR$\beta$ genes by a rearranged TCR$\beta$ transgene (L. Ardouin, J. Ismaili, B. Malissen, and M. Malissen. (1998) The CD3-$\gamma\delta\epsilon$ and CD3-$\zeta/\eta$ modules are each essential for allelic exclusion of the T cell receptor $\beta$ locus but are both dispensable for the initiation of V to (D)J recombination at the T cell receptor-$\beta$, -$\gamma$, and -$\delta$ loci. J. Exp. Med. 187:5). However, these as well as many previous studies on TCR$\beta$ transgene-mediated exclusion of endogenous TCR$\beta$ genes may have been misleading as recent work also suggested that this approach may generate transgenic artefacts (I. Aifantis, J. Buer, H. von Boehmer, and O. Azogui. (1997) Essential role of the pre-T cell receptor in allelic exclusion of the T cell receptor $\beta$ locus. Immunity 7: 601). Since much of the data on the role of *lck* in TCR$\beta$ allelic exclusion has been generated by this approach, it is possible that such transgenic artefacts have contributed to the inconsistencies concerning the role of *lck* in TCR$\beta$ locus allelic exclusion.

### BACKGROUND

During development in the thymus, $\alpha\beta$ lineage thymocytes proceed through two main developmental checkpoints: First, cells with a double coreceptor negative (DN) phenotype differentiate to double coreceptor positive (DP) cells; second, DP thymocytes develop into single coreceptor positive (SP)

cells (reviewed in Godfrey and Zlotnik, 1993). Progression through either of these checkpoints is the result of a process of selection (reviewed in Robey and Fowlkes, 1994). Differentiation of DN to DP thymocytes depends on the expression of the pre-TCR, consisting of the TCR$\beta$ chain, a surrogate TCR$\alpha$ polypeptide called pre-T$\alpha$, and components of the CD3 complex (Groettrup et al., 1993; Saint-Ruf et al., 1994). Differentiation of DP to SP cells depends on expression of the mature TCR, consisting of the TCR$\beta$ chain, together with a mature TCR$\alpha$ chain forming the TCR$\alpha\beta$ heterodimer, and the CD3 complex (reviewed in von Boehmer, 1990).

The TCR$\beta$ genes rearrange during the DN stage (Godfrey et al., 1994) and expression of the pre-TCR selects for the approximetely 56% of rearranging cells that statisticially succeed in generating a productive TCR$\beta$ gene (Mallick et al., 1993; Dudley et al., 1994). Selection by the pre-TCR, therefore, generates the repertoire of TCR$\beta$ chains (reviewed in Levelt and Eichmann, 1995). The TCR$\alpha$ genes rearrange during the DP stage (Petrie et al., 1993). DP cells expressing a mature $\alpha\beta$TCR are selected, positively or negatively, by the ability of the $\alpha\beta$TCR to interact with MHC/peptide ligands, thus generating the self-tolerant, self-MHC-restricted repertoire of mature $\alpha\beta$ T cells (reviewed in Rothenberg, 1994).

Pre-TCR-dependent maturation from DN to DP thymocytes is associated with a complex cluster of differentiation events (reviewed in Tanaka et al., 1995; Zunicka-Pflücker and Lenardo, 1996). To discuss all of its components would go far beyond the scope of a minireview. I will therefore concentrate on the two main developmental responses most relevant to the scope of this paper: (1) The burst of cell divisions: Induction of proliferation generates a large number of DP cells from each original TCR$\beta$-positive DN cell, thus giving rise to a diverse set of TCR$\beta$ clones for further diversification by combination with TCR$\alpha$ chains (Falk et al., 1996). (2) The transition from TCR$\beta$ to TCR$\alpha$ locus rearrangement: Once a DN cell has produced a TCR$\beta$ chain, signals through the pre-TCR arrest further rearrangements in the TCR$\beta$ locus (Levelt et al., 1995a), a process called allelic exclusion and believed to account for the fact that most T cells express only one of two possible TCR$\beta$ loci (Malissen et al., 1992). Shutdown of rearrangement is accompanied by transient down-regulation in the expression of the recombination activating genes RAG1 and RAG2 (Wilson et al., 1994), a response also induced through a pre-TCR-mediated signal (Levelt et al., 1995a). This is followed by initiation of TCR$\alpha$ locus germline transcription and initiation of rearrangement of the TCR$\alpha$ genes (Capone et al., 1993), a late consequence of pre-TCR-induced differentiation (Levelt et al., 1995a).

The function of the pre-TCR depends on intracellular signal transmission as suggested by the involvement of the p21$^{ras}$/MAP kinase pathway (Crompton et al., 1996). Moreover, the pre-TCR transmits its messages into the cell by signal transduction through the CD3 complex. This has first been suggested by experiments involving cross-linking of CD3$\epsilon$ on immature thymocytes by anti-CD3 antibodies (Levelt et al., 1993a). It was found that essentially all developmental responses ascribed to the pre-TCR could be induced by anti-CD3 (reviewed in Levelt and Eichmann, 1995). However, and unexpectedly, the immature thymocytes responding to anti-CD3 were in a developmental stage prior to expression of the TCR$\beta$ chain (Levelt et al., 1993a, 1993b). This suggested that CD3 components were expressed on thymocytes prior to the complete pre-TCR, but also raised doubts whether or not the antibody-induced signals were indeed a faithful reflection of the pre-TCR signals. Nevertheless, the involvement of CD3 signaling in the function of the pre-TCR has been further corroborated by the phenotype of mice genetically deficient in the CD3$\epsilon\delta\gamma$ components (Malissen et al., 1995). Thymocyte development in these mice is arrested at exactly the same stage as in mice that are unable to rearrange their TCR$\beta$ genes owing to deficiency in RAG1 or RAG2 (Mombaerts et al., 1992a; Shinkai et al., 1992).

Signaling through the CD3 complex critically depends on phosphorylation of immune-receptor tyrosine-activation motifs (ITAMs) (reviewed in Samelson and Klausner, 1992). ITAMs are associated with the cytoplasmic tails of CD3 components as well as of other receptors such as the lg$\alpha/\beta$ components of the B-cell receptor complex, and multiple forms of Fc receptors. The main family of protein tyrosine kinases (PTKs) involved in ITAM phosporylation in hemopoetic cells is the *src* family, consisting of nine members (reviewed in Cooper, 1990). As a general rule, members of the *src* family are in physical contact with cytoplasmic tails of members of the lymphocyte receptor complexes ((reviewed in Bolen et al., 1992; Chan et al., 1994; Chow and Veillette, 1995): The hemopoetic isoform of $59^{fyn}$ (*fynT*) is associated with members of the CD3 complex, and $p56^{lck}$ (*lck*) is associated with the coreceptors CD4 and CD8 on T cells. Similarly, $p53^{lyn}$ (*lyn*) and $p55^{blk}$ (*blk*), to a lesser extent also *lck* and *fynT*, are associated with the lg$\alpha/\beta$ components of the B-cell receptor. Upon receptor ligation, these PTKs phosporylate the ITAMs, which then act as docking sites for SH2 domains, thus recruiting PTKs belonging to the ZAP-70/Syk family into the signaling process. *Src*-family PTKs show preferential tissue-specific expression (reviewed in Chow and Veillette, 1995): The aforementioned *lck*, *fynT*, *lyn*, and *blk* are predominantly expressed in lymphocytes, $p58^{c\text{-}fgr}$ and $p56/59^{hck}$ are predominantly expressed in cells of the myeloid lineage, and $p60^{c\text{-}src}$, $p62^{c\text{-}yes}$, and perhaps also $p60^{yrk}$ are reported as ubiquitously expressed. Both lymphoid and myeloid *src*-PTKs have also been implicated in Fc-receptor and cytokine-receptor signal transduction.

Although the *src*-PTK *fynT* as well as ZAP-70 are intimately involved in CD3 signaling of mature thymocytes and T cells, targeted mutation of either PTK did not affect pre-TCR-dependent thymocyte differentiation (Appleby et al., 1992; Stein et al., 1992; Negishi et al., 1995). In contrast, genetic manipulations concerning *lck* had profound effects on several developmental responses ascribed to the pre-TCR. However, the defects observed in different genetic experiments were not always consistent with one another and with findings from other types of experiments. These inconsistancies give rise to the *lck* paradox and concern the two main components of pre-TCR-dependent differentiation: the burst of all divisions and the regulation of TCR gene rearrangements.

### *Lck* INVOLVEMENT IN PRE-TCR-INDUCED PROLIFERATION

Mice genetically unable to synthetize a complete pre-TCR fall into two different categories with respect to their impairment in pre-TCR-induced proliferation. One group, consisting of scid mice (Schuler et al., 1986), RAG1/2-deficient mice (Mombaerts et al., 1992a; Shinkai et al., 1992), and CD3$\gamma\delta\epsilon$-deficient mice (Malissen et al., 1995), is virtually completely defective, that is, its thymic cellularity remains at 1-2 $\times$ $10^6$ with almost no DP cells detected. The other group, consisting of TCR$\beta$-deficient mice (Mombaerts et al., 1992b), CD3$\xi$-deficient mice (reviewed in Tanaka et al., 1995), and pre-T$\alpha$-deficient mice (Fehling et al., 1995), has a residual proliferative activity generating 10-20 $\times$ $10^6$ thymocytes and about 10-20% of the normal number of DP cells. Some of the latter phenotypes have been explained by a positive feedback between thymic stroma and CD3-positive cells, that is, $\gamma\delta$ T cells (Shores et al., 1990, 1991; Ritter and Boyd, 1993). However, it is also possible that incomplete versions of the pre-TCR excert some residual signaling activity.

Two different genetic approaches toward the deletion of functional *lck* have been taken: Targeted mutation to delete the functional *lck* gene (Molina et al., 1992), and introduction of a transgene encoding a dominant negativ *lck* mutant that competes with the product of the wildtype *lck* gene (Levin et al., 1993). Paradoxically, mice carrying a dominant negative mutant *lck* gene fall into the category of strains with completely defective proliferation, whereas *lck*-deficient mice fall into the group with residual proliferative and differentiating activity.

Using the anti-CD3$\epsilon$ cross-linking approach, we have previously examined the proliferation defects in newborn RAG1/*lck* single- and double-deficient mice (Levelt et al., 1995b). RAG1-deficient mice increase their thymic cellularity from 1-2 $\times$ $10^6$ to 50 $\times$ $10^6$ cells after injection of anti-CD3. *Lck*-deficient mice show no change in their thymocyte numbers (10 × $10^6$) upon injection of anti-CD3. RAG1/*lck* double-deficient thymi increase from 1-2 × $10^6$ to 10 × $10^6$ cells after anti-CD3 treatment. These results suggest that the pre-TCR-dependent proliferative burst has two components; one appears to be *lck*-dependent, whereas the other may take place in the absence of *lck*. In RAG-deficient mice, CD3ε stimulation in the presence of *lck* initiates both components, whereas in the absence of *lck*, only the *lck*-independent component is stimulated. In *lck* single-deficient mice, the *lck*-independent component of pre-TCR-dependent proliferation takes place spontaneously, and anti-CD3 stimulation has no further effect. Although these results are consistent with one another, it remains obscure why a dominant negative mutant of *lck* apparently inhibits both the *lck*-dependent as well as the *lck*-independent component of pre-TCR-induced proliferation.

### *Lck* INVOLVEMENT IN THE INDUCTION OF TCRβ LOCUS ALLELIC EXCLUSION

Two experimental approaches have been used to assess competence of allelic exclusion of the TCRβ locus in mice. The first is based on the expression of a rearranged TCRβ transgene. In a normal mouse, this leads to the more or less complete inhibition of endogenous TCRβ V to DJ rearrangements, so that a majority of the thymocytes and peripheral T cells express only the transgenic TCRβ chain (Uematsu et al., 1988). The second approach to test competence of allelic exclusion is based on exposure of immature thymocytes to anti-CD3ε. Since immature thymocytes express CD3ε before they rearrange the TCRβ chain (Levelt et al., 1993a, 1993b), CD3 mediated signals delivered at that stage are misinterpreted by the cell as indicating the presence of a complete pre-TCR, including a TCRβ chain. As a result, the thymocyte arrests its rearrangement activity before it even started, resulting in a drastic reduction in the proportion of DP thymocytes expressing TCRβ chains (Levelt et al., 1993a). Arrest of rearrangement concerns the V to DJ step, just as observed for TCRβ transgenes (Levelt et al., 1995a).

Collectively, the data derived from TCRβ transgenes and from anti-CD3ε stimulation strongly support the notion that TCRβ locus allelic exclusion is controlled by stimulation through the pre-TCR. A pivotal role for *lck* in this control is suggested by the results from mice carrying a dominant negative *lck* mutant transgene, in which inhibition of endogenous TCRβ V to DJ rearrangements by a rearranged TCRβ transgene is abrogated (Anderson et al., 1993). Moreover, thymocytes from transgenic mice expressing a constitutively active mutant of *lck* showed a pronounced diminution of TCRβ V to DJ rearrangements (Anderson et al., 1992). However, in contrast to these results, little or no defectiveness in the inhibition of endogenous TCRβ V to DJ rearrangements by a rearranged TCRβ transgene was seen in mice in which the *lck* gene has been deleted by targeted mutation (Wallace et al., 1995). Upon quantitative analysis, the proportion of thymocytes with endogenous TCRβ chains in TCRβ transgenic *lck*-deficient mice did not exceed 10% of that seen in the absence of the TCRβ transgene. Thus, more so than in pre-TCR-dependent proliferation, the *in vivo* effects of a dominant negative mutant of *lck* and of the deletion of the *lck* gene are paradoxically discrepant. An additional inconsistency becomes apparent on attempts to inhibit TCRβ locus rearrangement in *lck*-deficient mice by injection of anti-CD3ε antibody. In multiple experimental attempts, no suppression of TCRβ locus rearrangement could be induced (C.N. Levelt and K. Eichmann, unpublished observations). These findings tend to support the results obtained with the dominant negative *lck* mutant and contradict that of a TCRβ transgene in *lck*-deficient mice.

The situation created by these conflicting data is truely paradoxical as no reasonably straightforward hypothesis can resolve all inconsistencies. For example, let us consider the hypothesis that the pre-TCR can signal along two different pathways, a major one involving CD3ε and *lck*, and a minor one utilizing CD3ξ and other *src* PTKs, for example, *fynT*. If we assume that the dominant negative mutant *lck* successfully competes not only with *lck*, but also with *fynT*, the discrepancy between the drastic effects of the dominant negative mutant *lck* and the mild effects of *lck* deficiency could be readily explained. As far as allelic exclusion is concerned, the discrepancy between the effects of a TCR$\beta$ transgene and of anti-CD3$\epsilon$ stimulation in *lck*-deficient mice are resolved by this model if one considers that anti-CD3 stimulation triggers thymocytes before assembly of a complete pre-TCR, whereas a TCR$\beta$ transgene exerts its effect through a complete pre-TCR. Before assembly of the pre-TCR, CD3 components are present as independent $\epsilon\delta$ and $\epsilon\gamma$ dimers, presumably not associated with CD3$\xi$ (Wiest et al., 1994). Thus, anti-CD3$\epsilon$ cross-linking would activate the CD3$\epsilon$-*lck* pathway but not the CD3$\xi$-*fynT* pathway, and therefore would be ineffective in *lck*-deficient mice. Conversely, a complete pre-TCR is functionally associated with the CD3$\xi$ chain (Vanoers et al., 1995) and therefore can induce allelic exclusion via *fynT* even in *lck*-deficient mice. So far, this hypothesis seems quite satisfactory. However, within this model, stimulation of proliferation of RAG1/*lck* double-deficient thymocytes by anti-CD3$\epsilon$, which clearly takes place (Levelt et al., 1995b), should not be possible. The reader is encouraged to try alternative models; all of my own attempts have been unsuccessful.

It should be pointed out that even if the *lck* paradox could be resolved, pre-TCR control alone seems insufficient to explain all present data on the regulation of allelic exclusion. In a RAG2 complementation approach, ES cells homozygously deleted for pre-T$\alpha$ differentiated into thymocytes in which inhibition of endogeneous TCR$\beta$ rearrangements by a TCR$\beta$ transgene was fully maintained (Xu et al., 1996). This is strikingly reminiscent of IgH-chain allelic exclusion in B cells of mice deficient of the surrogate L chain of the pre-B-cell receptor: Although immature B cells in these mice were defective in allelic exclusion, mature B cells showed fully maintained allelic exclusion (Löffert et al., 1996). Thus, allelic exclusion in either B or T cells may be protected by two safeguards, one provided by signaling through the preT/B-cell receptor complexes, the other by a downstream developmental checkpoint preventing the maturation of cells that have failed to become allelically excluded.

### References

Anderson S.J., Abraham K.M., Nakayama T., Singer A., and Perlmutter R.M. (1992). Inhibition of T-cell receptor $\beta$-chain gene rearrangement by overexpression of the non-receptor protein tyrosine kinase p56$^{lck}$. EMBO J. **11**: 4877-4886.

Anderson S.J., Levin S.D., and Perlmutter R.M. (1993). Protein tyrosine kinase p56$^{lck}$ controls allelic exclusion of T-cell receptor $\beta$ chain genes. Nature **365**: 552-554.

Appleby M.W., Gross J.A., Cooke M.P., Levin S.D., Qian X., and Perlmutter R.M. (1992). Defective T cell receptor signaling in mice lacking the thymic isoform of p59$^{fyn}$. Cell **70**: 751-763.

Bolen J.B., Rowley R.B., Spana C., and Tsygankov A.Y. (1992). The *src*-family of tyrosine protein kinases in hemopoietic signal transduction. FASEB J. **6**: 3403-3409.

Capone M., Watrin F., Fernex C., Horvat B., Krippl B., Wu L., Scollay R., and Ferner P. (1993). TCR $\beta$ and TCR $\alpha$ gene enhancers confer tissue- and stage-specificity on V(D)J recombination events. EMBO J. **12**: 4335-4346.

Chan A.C., Desai D.M., and Weiss A. (1994). The role of protein tyrosine kinases and protein tyrosine phosphatases in T cell antigen receptor signal transduction. Annu. Rev. Immunol. **12**: 555-592.

Chow L.M.L., and Veillette A., (1995). The src and csk families of tyrosine protein kinases in hemopoetic cells. Sem. Immunol. **7**: 107-226.

Cooper J.A. (1990). The *src*-family of protein-tyrosine kinases. In Peptides and Protein Phosphorylation, Kemp B.E. Ed. (Boca Raton, FL: CRC Press), pp. 85-115.

Crompton T., Gilmour K.C., and Owen M.J. (1996). The MAP kinase pathway controls differentiation of double-negative to double-positive thymocyte. Cell **86**: 243-251.

Dudley E.C., Petrie H.T., Shah L.M., Owen M.J., and Hayday A.C. (1994). T cell receptor $\beta$ chain gene rearrangement and selection during thymocyte development in adult mice. Immunity **1**: 83-92.

Falk I., Biro J., Kohler H., and Eichmann K. (1996). Proliferation kinetics associated with T-cell-receptor $\beta$ chain selection of fetal murine thymocytes. J. Exp. Med. **184**: 2327-2339.

Fehling J.J., Krotkova A., Saint-Ruf C., and von Boehmer H. (1995). Crucial role of the pre-T cell receptor $\alpha$ gene in the development of $\alpha\beta$ but not of $\gamma\delta$ cells. Nature **375**: 795-798.

Godfrey D.I., Kennedy J., Mombaerts P., Tonegawa S., and Zlotnik A. (1994). Onset of TCR$\beta$ rearrangment and role of TCR$\beta$ expression during CD3$^-$CD4$^-$CD8$^-$ thymocyte differentiation. J. Immunol. **152**: 4783-4792.

Godfrey D.I., and Zlotnik A. (1993). Control points in early T-cell development. Immunol. Today **14**: 547-554.

Groettrup M., Ungewiss K., Azogui O., Palacios R., Owen M.J., Hayday A.C., and von Boehmer H. (1993). A novel disulphide-linked heterodimer on pre-T cells consists of the T cell receptor $\beta$ chain and a 33 kD glycoprotein. Cell **75**: 283-294.

Levelt C.N., Ehrfeld A., and Eichmann K. (1993a). Regulation of thymocyte development through CD3. I. Timepoint of ligation of CD3$\epsilon$ determines clonal deletion or induction of developmental program. J. Exp. Med. **177**: 707-716.

Levelt C.N., and Eichmann K. (1995). Receptors and signals in early thymic selection. Immunity **3**: 667-672.

Levelt C.N., Mombaerts P., Iglesias A., Tonegawa S., and Eichmann K. (1993b). Restoration of early thymocyte differentiation in T-cell receptor $\beta$-chain deficient mutant mice by transmembrane signaling through CD3$\epsilon$. Proc. Natl. Acad. Sci. USA **90**: 11401-11405.

Levelt C.N., Mombaerts P., Wang B., Kohler H., Tonegawa S., Eichmann K., and Terhorst C. (1995b). Regulation of thymocyte development through CD3: Functional dissociation between $p56^{lck}$ and CD3ζ in early thymic selection. Immunity **3**: 215-222.

Levelt C.N., Wang B., Ehrfeld A., Terhorst C., and Eichmann K. (1995a). Regulation of T cell receptor (TCR)-β locus allelic exclusion and initiation of TCRα locus rearrangement in immature thymocytes by signaling through the CD3 complex. Eur. J. Immunol. **25**: 1257-1261.

Levin S.D., Anderson S.J., Forbush K.A., and Perlmutter R.M. (1993). A dominant-negative transgene defines a role for $p56^{lck}$ in thymopoiesis. EMBO J. **12**: 1671-1680.

Löffert D., Ehlich A., Müller W., and Rajewsky K. (1996). Surrogate light chain expression is required to establish immunoglobulin heavy chain allelic exclusion during early B cell development. Immunity **4**: 133-144.

Malissen M., Gillet A., Ardouin L., Bouvier G., Trucy J., Ferrier P., Vivier E., and Malissen B. (1995). Altered T cell development in mice with a targeted mutation of the CD3ε gene. EMBO J. **14**: 4641-4653.

Malissen M., Tracy J., Janvin-Marche E., Cazenave P.A., Scollay R., and Malissen B. (1992). Regulation of TCRα and β gene allelic exclusion during T cell development. Immunol. Today **13**: 315-322.

Mallick C.A., Dudley E.C., Viney J.L, Owen M.J., and Hayday A.C. (1993). Rearrangement and diversity of T cell receptor β chain genes in thymocytes, a critical role for the β chain in development. Cell **73**: 513-519.

Molina T.J., Kishihara K., Siderovski D.P., Ewijk W., van Narendran A., Timms E., Wakeham A., Paige C.J., Hartmann K.-U., Veillette A., Davidson D., and Mak T.W. (1992). Profound block in thymocyte development in mice lacking $p56^{lck}$. Nature **357**: 161-164.

Mombaerts P., Clarke A.R., Rudnicki M.A., Iacomini J., Itohara S., Lafaille J.J., Wang L., Ichikawa Y., Jaenisch R., Hooper M.L., and Tonegawa S. (1992b). Mutations in T cell antigen receptor genes α and β block thymocyte development at different stages. Nature **360**: 225-231.

Mombaerts P., Iacomini J., Johnson R.S., Herup K., Tonegawa S., and Papaicannou V.E. (1992a). RAG-1-deficient mice have no mature B and T lymphocytes. Cell **68**: 869-877.

Negishi I., Motoyama N., Nakayama K.-I., Nakayama K., Senju S., Hatakeyama S., Zhang Q., Chan A.C., and Loh D.Y. (1995). Essential role for ZAP-70 in both positive and negative selection of thymocytes. Nature **376**: 435-438.

Petrie T., Livak F., Schatz D.G., Strasser A., Crispe I.N., and Shortman K. (1993). Multiple rearrangements in T cell receptor α chain genes maximize the production of useful thymocytes. J. Exp. Med. **178**: 615-622.

Ritter M.A., and Body R.L. (1993). Development in the thymus: it takes two to tango. Immunol. Today **14**: 462-469.

Robey E., and Fowlkes B. J. (1994). Selective events in T cell development. Ann Rev. Immunol. **12**: 675-705.

Rothenberg E.V. (1994). Signaling mechanisms in thymocyte selection. Curr. Opin. Immunol. **6**: 257-265.

Saint-Ruf C., Ungewiss K., Groettrup M., Bruno L., Fehling H.L., and von Boehmer H. (1994). Analysis and expression of a pre-T cell receptor gene. Science **266**: 1208-1212.

Samelson L.E., and Klausner R.D. (1992). Tyrosine kinases and tyrosine-based activation motifs. J. Biol. Chem. **267**: 24913-24916.

Stein P.L., Lee H.-M., Rich S., and Soriano P. (1992). $pp59^{fyn}$ mutant mice display differential signaling in thymocytes and peripheral T cells. Cell **70**: 741-750.

Schuler W., Weiler I.J., Schuler A., Philips R.A., Rosenberg N., Mak T.W., Kearney J.F., Perry R.P., and Bosma M.J. (1986). Rearrangement of antigen receptor genes is defective in mice with severe combined immune deficiency. Cell **46**: 963-972.

Shinkai Y., Rathbun G., Lam K.P., Oltz E.M., Stewart V., Mendelsohn M., Charron J., Datta M., Young F., Stall A.M., and Alt F.W. (1992). RAG2-deficient mice lack mature lymphocytes owing to inability to initiate V(D)J rearrangement. Cell **68**: 855-867.

Shores E.W., Sharrow S.O., Uppenkamp I., and Singer A. (1990). T cell receptor negative thymocytes from Scid mice can be induced to enter the CD4/CD8 differentiation pathway. Eur. J. Immunol. **20**: 69-75.

Shores E.W., van Ewijk W., and Singer A. (1991). Disorganization and restoration of thymic medullary epthelial cells in T cell receptor-negative scid mice: Evidence that receptor-bearing lymphocytes influence maturation of the thymic environment. Eur. J. Immunol. **21**: 1657-1661.

Tanaka Y., Ardouin L., Gillet A., Lin S.Y., Magnan A., Malissen B., and Malissen M. (1995). Early T-cell development in CD3-deficient mice. Immunol. Rev. **148**: 171-199.

Uematsu Y., Ryser S., Dembic Z., Borgulya P., Krimpenfort P., Berns A., von Boehmer H., and Steinmetz M. (1988). In transgenic mice the introduced functional T cell receptor β gene prevents expression of endogenous β genes. Cell **52**: 831-841.

Von Boehmer H. (1990). Developmental biology of T cells in T cell-receptor transgenic mice. Annu. Rev. Immunol. **8**: 531-556.

Vanoers N.S.C, von Boehmer H., and Weiss A. (1995). The pre-T-cell receptor (tcr) is functionally coupled to the tcr-zeta subunit. J. Exp. Med. **182**: 1585-1590.

Wallace V.A., Kawai K., Levelt C.N., Kishihava K., Molina T., Timms E., Pircher H., Penninger J., Ohashi P.S., Eichmann K., and Mak T.W. (1995). T lymphocyte development in $p56^{lck}$ deficient mice: Allelic exclusion is incomplete but thymocyte development is not restored by TCRβ or TCRαβ transgenes. Eur. J. Immunol. **25**: 1312-1318.

Wiest D.L., Kearse K.P., Shores E.W., and Singer A. (1994). Developmentally regulated expression of CD3 components independently of clonotypic T cell antigen receptor complexes on immature thymocytes. J. Exp. Med. **180**: 1375-1382.

Wilson A., Held W., and MacDonald H.R. (1994). Two waves of recombinase gene expression in developing thymocytes. J. Exp. Med. **179**: 1355-1360.

Xu Y., Davidson L., Alt F.W., and Baltimore D. (1996). Function of the pre-T-cell receptor α chain in T cell development and allelic exclusion at the T-cell receptor β-locus. Proc. Nat. Acad. Sci. USA **93**: 2169-2173.

Zunicka-Pflücker J.L., and Lenardo M.J. (1996). Regulation of thymocyte development from immature progenitors. Curr. Op. Immunol. **8**: 215-224.

